# The role of lipid metabolism in tumor immune microenvironment and potential therapeutic strategies

**DOI:** 10.3389/fonc.2022.984560

**Published:** 2022-09-12

**Authors:** Danting Wang, Qizhen Ye, Haochen Gu, Zhigang Chen

**Affiliations:** ^1^ Department of Breast Surgery (Surgical Oncology), Second Affiliated Hospital, Zhejiang University School of Medicine, Hangzhou, China; ^2^ Key Laboratory of Tumor Microenvironment and Immune Therapy of Zhejiang Province, Second Affiliated Hospital, Zhejiang University, Hangzhou, China; ^3^ Cancer Centre, Zhejiang University, Hangzhou, China; ^4^ Cancer Institute, Key Laboratory of Cancer Prevention and Intervention, Ministry of Education, The Second Affiliated Hospital, Zhejiang University School of Medicine, Hangzhou, China

**Keywords:** lipid metabolism, tumor, immune cells, immunotherapy, microenvironment

## Abstract

Aberrant lipid metabolism is nonnegligible for tumor cells to adapt to the tumor microenvironment (TME). It plays a significant role in the amount and function of immune cells, including tumor-associated macrophages, T cells, dendritic cells and marrow-derived suppressor cells. It is well-known that the immune response in TME is suppressed and lipid metabolism is closely involved in this process. Immunotherapy, containing anti-PD1/PDL1 therapy and adoptive T cell therapy, is a crucial clinical cancer therapeutic strategy nowadays, but they display a low-sensibility in certain cancers. In this review, we mainly discussed the importance of lipid metabolism in the formation of immunosuppressive TME, and explored the effectiveness and sensitivity of immunotherapy treatment by regulating the lipid metabolism.

## Introduction

Lipids are hydrophobic molecules including phospholipids, monoglycerides, diacylglycerides, triglycerides and sterols, which play the function of participating in forming biological membranes, providing energy, storing energy and mediating cellular signal transduction. Among them fatty acids (FAs), defined as a diverse class of molecules consisting of hydrocarbon chains varying in length and saturation, indispensably involve in the lipid metabolism which includes exogenous lipid uptake, *de novo* lipid synthesis and lipolysis (1). Fatty acid oxidation (FAO, also called beta-oxidation), shortens fatty acids by two carbons in each cycle and generates NADH, FADH2 and acetyl CoA through its series of cyclic reactions. And the end product of FAO pathway, acetyl CoA, can be ultimately converted back to fatty acids through the catalysis of various enzymes in the process of FA synthesis (FAS). Besides, cholesterol, which deemed as an indispensable part of biological membranes and the substrate for steroid hormones, is also derived from acetyl CoA and its synthesis is inseparable from many enzymes like Acyl coenzyme A-cholesterol acyltransferase (ACAT) (2).The above lipid metabolic processes are of great significance and studies revealed that lipid metabolism has undergone a variety of metabolic reprogramming in tumor cells to promote tumor growth, proliferation and metastasis (3, 4), and the diversity of lipid types and their different roles in tumor cells lead to the complex mechanism alterations. Therefore, targeting lipid metabolism is a promising strategy for cancer treatment. (5).

It is inadequate for tumor cells to rely solely on glucose metabolism for energy. Therefore, in order to meet the need of rapid growth and proliferation, tumor cells promote lipid metabolism through a series of mechanisms. Firstly, higher expression of enzymes related to lipid uptake such as CD36 enables tumor cells to compete with other cells for lipids from TME. Secondly, transcription factors related to lipid oxidation enzymes have been found to be expressed at high frequency. Thirdly, tumor cells have the ability to synthesize lipids independently. In addition to providing energy, lipids also serve as signal transduction molecules and raw materials of cellular structure in tumor cells (6). Furthermore, cholesterol and phospholipid biosynthesis are also very active in tumor cells, they maintain the integrity and fluidity of cell membranes to ensure the survival of tumor cells, and affect the function of receptors on the membrane to enable tumor cells' drug-resistance and better adaptation to the TME (7).

In the hypoxic and acidic TME, dysfunctional immune cells including tumor-associated macrophages (TAMs), tumor infiltrating T cells (TILs), dendritic cells (DC), myeloid-derived suppressor cells (MDSC), and natural killer cells (NK) lead to muted immune responses and further promote tumor progression (8). There is also a fierce competition for the deficient nutrients between immune cells and tumor cells in the TME, during which process their lipid metabolic reprogramming are of great significance and closely involved in the immunosuppressive outcome (9, 10). Pro-tumor and anti-tumor immune cells have different levels of lipid metabolism activity, the former, such as M2-macrophages and Treg cells, tend to have more active lipid metabolism, while the latter prefer to acquire energy through glucose metabolism (11). This difference in lipid metabolism is regulated by many factors, especially some transcriptional regulators such as peroxisome proliferator-activated receptor (PPAR) and sterol regulatory element-binding proteins (SREBP) (12). These lipid metabolism reprogramming in immune cells together leads to the formation of the immunosuppressive microenvironment.

Immunotherapy including adoptive cell therapy, immune checkpoint blockade (ICB), tumor-specific vaccines and small-molecule immune drugs has shown certain therapeutic effects. As one of the representatives of immunotherapy, programmed cell death protein 1 (PD-1) antibodies can inactivate tumor cells’ checkpoint and unleash cytotoxic T cells’ anti-tumor effects, but still major patients do not show complete responses (13). At present, tumor immunotherapy still faces many challenges, and it has a beneficial effect on the minority of patients (14). The immunotherapy resistance and post-immunotherapy cancer progression are two main problems accounting for that low-sensitivity (15, 16). Since lipid metabolism plays an important role in the function of immune cells, there are many experiments to improve the function of immune cells by regulating lipid metabolism, so that immunotherapy may be achieve better efficacy. Our review summarizes and analyzes existing studies with clinical application value, providing useful references for researchers’ follow-up research. For more precise targeted treatment, future research needs to focus on the sensitivity of regulating the same lipid metabolism target in different types of immune cells. Combining lipid metabolism regulation with immunotherapy may lead to new breakthroughs in immunotherapy.

## Lipid metabolism reprogramming in tumor cells and tumor stromal cells

**Figure 1:**
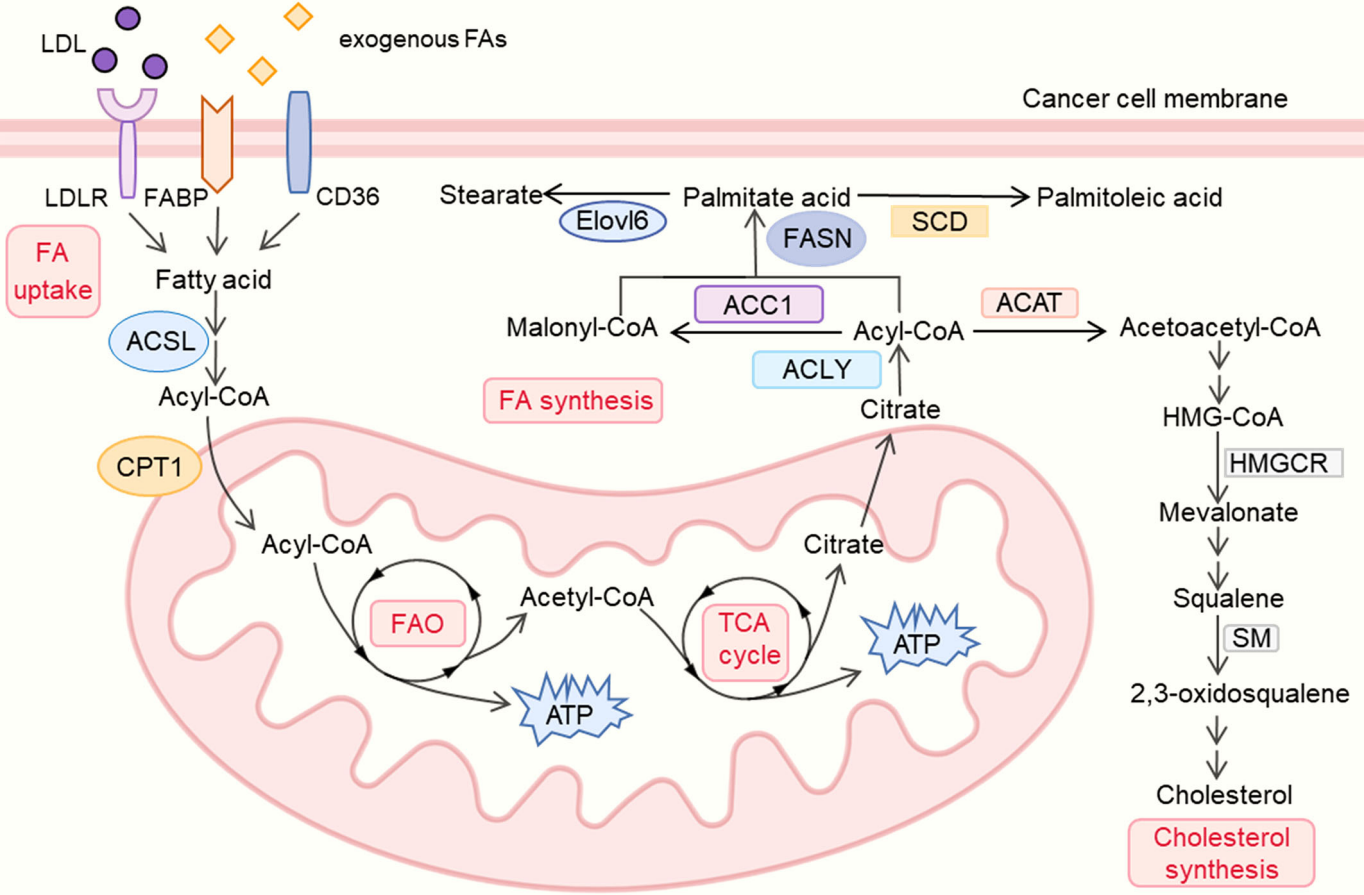
lipid metabolism reprogramming in cancer cells. LDL, low density lipoprotein; LDLR, low density lipoprotein receptor; FA, fatty acid; FABP, fatty acid binding protein; ACSL, Acyl-CoA synthetase long chain; CPT, carnitine palmitoyl transferase; FAO, fatty acid oxidation; TCA, tricarboxylic acid cycle; ACLY, ATP–citrate lyase; ACC, Acetyl-CoA carboxylase; FASN, fatty-acid synthase; SCD, Stearoyl-CoA desaturase; Elovl6, Elongation of long-chain fatty acids family member 6; ACAT, Acyl coenzyme A-cholesterol acyltransferase; HMGCR, 3-hydroxy-3-methylglutaryl coenzyme A reductase; SM, squalene monooxygenase.

As a feature of many malignant tumors, lipid metabolic reprogramming, especially the dynamic balance between FAS and FAO, plays a significant role in tumor progression (1) (**Figure 1**).

Studies have shown that lipogenesis plays an indispensable part in the growth of numerous tumors (17). On the one hand, lipids from the extracellular milieu are crucial for tumor cells. Studies found that tumors can acquire exogenous FAs like palmitic acid which was proved to promote metastasis in carcinoma (18). The upregulation of multiple FA protein transporters, particularly low-density lipoprotein receptors (LDLRs), CD36 (fatty acid translocase) and fatty acid-binding proteins (FABPs), were found in various tumors’ plasma membranes. Low density lipoproteins (LDLs) can be taken in *via* the LDLRs on the membrane, and the high expression of LDLRs promotes the LDL cholesterol-mediated breast cancer growth (19), which is also associated with the poor prognosis in small cell lung cancer (SCLC) and ovarian cancer (OC) (20, 21). High expression of CD36, which has been correlated with poor survival for patients with gastric, ovarian and breast cancer, could enhance the uptake of diet-derived FAs and fuel tumors’ growth and metastasis (22, 23). Besides, elevated FAs imported *via* CD36 were also testified to accelerate tumors’ epithelial-mesenchymal transition (EMT) (24), while inhibition of CD36 could impair the metastasis of human oral cancers and prevent the development of the adipocyte-induced malignant phenotypes in ovarian cancer (18, 25). Additionally, FABPs also play an instrumental role in cancer by affecting fatty acid metabolism, high expression of epidermal FABP (E-FABP) in the TAMs could enhance the production of interferon-beta (IFN-β) against the growth of tumor (26). The above evidence indicated the diversity of FA protein transporters’ functions in tumor therapy and suggested potential treatments such as dietary regulation in further study.

Apart from enhancing uptake of exogenous FAs, tumors also exhibit a high level of *de novo* FA synthesis which alters cellular lipid composition and fuels tumor growth (27). This process involves the upregulation of many enzymes as follows. Acetyl–coenzyme A (CoA), as the main substrate of lipid synthesis, can be converted from citrate by ATP–citrate lyase (ACLY). Since the production of citrate could be derived from glucose (22), ACLY is also regarded as a key enzyme linking glycolysis and lipid synthesis. High ACLY expression was found in gastric adenocarcinoma patients, correlated with advanced stages and lymph node metastasis (28), ACLY also shows a strong association with poor prognosis in various cancers like ovarian cancer (29). On the contrary, the ACLY-targeted treatments exhibit tumor- suppressive effects. ACLY inhibitor SB-204990 could not only suppress lipid synthesis and tumor progression but also show a stronger effect in combination therapy with cisplatin by inhibiting the PI3K-AKT pathway and activating the AMPK-ROS pathway (29, 30).

Acetyl-CoA carboxylase (ACC), which catalyzes the carboxylation of acetyl-CoA to malonyl-CoA, was also found highly expressed in multiple tumors like breast cancer (31), ACC includes two isoforms, the cytosolic ACC1 is present in lipogenic tissues and is critical for FA synthesis, while ACC2 is bound in the mitochondrial outer membrane, mainly presenting in lipid oxidizing tissues like skeletal muscles. ACC1 promotes the FAO‐mediated human hepatocellular carcinoma (HCC) survival under conditions of metabolic stress and also contributes to the metastasis and recurrence of breast cancer (32, 33). While using RNA interference (RNAi) to silence the ACC-α gene could inhibit cell proliferation and induce apoptosis of prostate cancer cells (34). Unlike ACC1, ACC2 plays a role in inhibiting lipid degradation and was found inhibited in various cancers, its expression is also negatively correlated with tumor size and clinical stages of lung adenocarcinoma patients (35, 36). Prolyl hydroxylase domain protein 3 (PHD3) could also repress FAO and inhibit leukemia cell proliferation by activating ACC2 (37). The evidence revealed that both ACC1 and ACC2 could be potential targets in the treatment of cancer.

Fatty-acid synthase (FASN) is a multi-enzyme protein complex which can convert one acetyl-CoA molecule and seven malonyl-CoA molecules into one palmitate acid (C16: 0). FASN was found highly expressed in multiple cancers and closely related to poor prognosis, and notably, many studies have revealed that the downregulation of FASN can repress tumor progression *via* diverse mechanisms (3). Inhibition of FASN could not only lead to the accumulation of malonyl-CoA, which further represses tumor cells’ FAO and ultimately blocks the cell-cycle, but also impair the correct localization and/or functioning of EGFR and ERBB2 (3, 38). Besides, the apoptosis of tumor cells is also attributed to the inhibition of FASN which causes the downregulation of Akt and the starvation of phosphatidylcholine (3, 39). In terms of combination therapies, FASN inhibition with TVB-3166 or TVB-3664 could inhibit tubulin palmitoylation and show stronger anti-tumor effects with the combination of taxane drugs (40). And another selective FASN inhibitor, Fasnall, also reduces tumor volumes and extends survival when combined with carboplatin in the MMTV-Neu model of HER2^+^ breast cancer (41). AS a druggable target for cancer treatment, FASN has shown efficacy in diverse tumors, and its relevant metabolic treatments have broad prospects.

Palmitate acid (16:0) produced in *de novo* FA synthesis can be desaturated to palmitoleic acid (16:1) by Stearoyl-CoA desaturase (SCD). Since the desaturation driven by SCD1 could enhance tumor intrinsic antioxidants and anti-ferroptotic resources for survival and regrowth, suppression of SCD1 was also found to show anti-tumor effects (42). Researchers revealed the association between high expression of SCD1 and lung adenocarcinoma patients’ late stage, which provides SCD1 as a novel potential therapeutic target to suppress tumors (43). Apart from being desaturated by SCDs, palmitate acid could also be elongated by the Elongation of long-chain fatty acids family member 6 (Elovl6) which is also associated with poor prognosis in patients with HCC and breast cancer (44, 45). Knockdown of Elovl6 could also reduce HCC cell proliferation and Akt activation, which further represses tumor growth, and could be used as a potential therapeutic approach (44). High expression of various enzymes which enhances *de novo* lipid synthesis in tumor cell is also deemed as predictors or biomarkers, and the downregulation of these enzymes shows its anti-tumor effects in different cancers, which reveals the significance of drugs targeting lipid synthesis in tumor therapy.

Tumor cells also exhibit a high level of FAO to meet their demands for energy for rapid proliferation. Since the acidosis in the TME causes the reduction of glucose-derived acetyl-CoA, FAO (also called beta-oxidation), which converts long-chain FAs into acetyl-CoA and meanwhile generates NADH and FADH_2_ for electron transport chain, plays an essential role in the energy production of tumors (1). Overexpression of FAO enzymes can also be found in multiple tumors like breast cancer, colorectal cancer and gastric cancer (46–48). As the rate-limiting enzyme in FAO, carnitine palmitoyl transferase 1 (CPT1) can transport acetyl-CoA to the mitochondrial matrix and CPT1 includes three isozymes who have different distributions and close relations with tumor. CPT1A is widely distributed in liver, pancreas, brain and blood, and it is also highly correlated with poor prognosis in acute myeloid leukemia (AML) or ovarian cancer (22). CPT1A could also support Castration-Resistant Prostate Cancer (CRPC) by supplying acetyl groups for histone acetylation and CPT1A variant 2 is also found to promote tumor invasion and metastasis (49, 50) Besides, knockdown of CPT1A could repress xenograft tumor initiation through inhibiting adipocytes’ tumor-promoting effects, which indicates CPT1A-dependent FAO as a target for anti-cancer therapeutics (51). CPT1B is selectively present in brown adipose tissue, muscle and heart, JAK/STAT3 induces the expression of CPT1B and further enhances FAO activity to promote breast cancer chemoresistance and stem cells self-renewal, while inhibiting the STAT3-CPT1B-FAO pathway could recover tumor’ sensitivity to chemotherapy (52). Unlike CPT1A and CPT1B, CPT1C is primarily present in the brain and has oncogenic potential (53). Research found CPT1C was remarkably reduced in senescent cells, and silencing of CPT1C could also cause cellular senescence in tumor and suppress xenograft tumor growth (54). CPT1C is also deemed as a contributor to tumor cell metabolic transformation and rapamycin resistance, which is significant for the growth of tumor cells under the conditions of metabolic stress (53).

Acyl-CoA synthetase long chain 3 (ACSL3), an upstream enzyme of CPT1, which converts free FAs into fatty acyl-CoAs, is also crucial for the proliferation of KRAS-driven cancer cells (55). Studies found that ACSL3 is upregulated in human pancreatic ductal adenocarcinoma (PDAC), and the deletion of ACSL3 could delay PDAC progression and reduce fibrosis in mice (56). Apart from energy production, FAO also generates nicotinamide adenine dinucleotide phosphate (NADPH) which is derived from IDH (isocitrate dehydrogenase)-mediated isocitrate oxidation to maintain homeostasis, which is also regarded as a potential therapeutic target (4). Using etomoxir to decrease NADPH level could result in cell death in human glioblastoma cells accompanied by oxidative stress (57). Taken together, the up-regulation of FAS and FAO levels in tumor cells meets the energy need of their rapid proliferation, many enzymes play significant roles in this process, and the down-regulation or inhibitors of those enzymes have been found to have anti-tumor effects with multiple mechanisms. Still some enzymes whose correlation with tumor and potential therapeutic effects remain to be confirmed, but the metabolic regulation related to lipid metabolism is no doubt playing an indispensable role in this field.

Multiple studies suggested that lipids other than fatty acids, such as cholesterol, also play a key role in tumor progression, and the inhibition of cholesterol synthesis is detrimental to cancer cells (6, 58). Cholesterol biosynthesis begins with the formation of acetoacetyl-CoA by the condensation of two molecules of acetyl coenzyme A through Acyl co-enzyme A-cholesterol acyltransferase (ACAT). And the inhibition of ACAT could reduce the formation of lipid droplets (LD) which further suppresses glioblastoma growth (59). Meanwhile, ACAT inhibitor such as avasimibe, was also proved to induce cell cycle arrest and apoptosis, which is regarded as a potential therapy in the treatment of glioblastoma (60).

As the rate-limiting enzyme in cholesterol biosynthesis, 3-hydroxy-3-methylglutaryl coenzyme A reductase (HMGCR) which reduces 3-hydroxy-3-methylglutaryl-CoA (HMG-CoA) to mevalonate was also found up-regulated in various cancers. Its overexpression induces statin resistance of cancer cells and accelerates tumor migration, while HMGCR knockdown could restore the statin sensitivity of tumor cells like breast cancer and CRPC (61, 62). In addition, HMGCR inhibitor simvastatin could also robustly enhances cancer vaccinations and synergizes with anti-PD-1 antibodies, which shows the broad therapeutic perspectives of drugs targeting cholesterol pathways in combination with other anti-tumor treatments (63). Apart from HMGCR, squalene monooxygenase (SM, also known as squalene epoxidase) which converts squalene to 2,3(S)-oxidosqualene is deemed as the second rate-limiting enzyme in cholesterol biosynthesis and its increased expression was also found associated with poor prognosis in several tumors like prostate cancer, pancreatic adenocarcinoma (PAAD) and head and neck squamous cell carcinoma (HNSCC) (64–66). While inhibition of SM could lead to the accumulation of squalene which further suppresses SCLC proliferation (67), and its pharmacological blockage also inhibits the invasion of CRPC (68). The above evidence reveals the potential value of various enzymes in cholesterol metabolism in tumor therapy, suggesting new ideas for tumor treatment.

When it comes to phospholipid metabolism, the extracellular choline and ethanolamine are transported into tumor cells to be catalyzed by a series of enzymes such as ethanolamine kinase (ETNK), choline kinase (ChK), cytidylyltransferase (CT), diacylglycerol phosphotransferase (PT), and respectively converted into phosphatidylcholine (PtdCho) and phosphatidylethanolamine (PtdEth) (69, 70). Metabolic remodeling of phospholipids can promote tumor progression and drug resistance. In glioblastoma (GBM), overexpressed polymerase 1 and transcript release factors (PTRF) lead to increased activity of cytoplasmic phospholipase A2 (cPLA2), and PTRFs can also induce decreased infiltration of CD8+ T cells. Ultimately, they promote the proliferation of tumor cells (71). Since phospholipid and cholesterol are the main raw materials for forming plasma membrane, their composition changes affect the activity and function of drug efflux pump on plasma membrane, and make tumor cells have stronger drug resistance to some extent (7).

Cancer-associated fibroblasts (CAF) are the major components of tumor stromal cells in TME. CAF-derived exosomes contain a variety of metabolites, including lipids, amino acids, and TCA cycle products (72), which play a major role in regulating various metabolic reprogramming in the tumor microenvironment, also including lipid metabolism (73). These products enter the TME and serve as an important source of extracellular lipids uptake by tumor cells and immune cells. Studies have shown that FAs or lysophosphatidylcholine (LPCs) overexpressed in fibroblasts can be released into TME, which subsequently are partially taken up by tumor cells and promote tumor growth through intracellular lipid metabolism reprogramming pathways (74, 75). Similar experiments have shown that CAF transport proteins and lipids to tumor cells in a one-way process (76). Moreover, CAFs induce the increased expression of fatty acid transport protein 1 (FATP1) in breast cancer cells and accelerate lipid uptake (77). CAFs in lung induce high expression of SCD1 in tumor cells through PI3K/Akt/mTOR pathway, which enhances the ability of tumor cells to metastasize and may be related to lung metastasis of colon cancer or breast cancer (74, 78). Meanwhile, CAFs contribute to promoting immune evasion and are the core source of immunosuppressive molecules. The resistance to ICB is also strictly controlled by CAFs (79). There is evidence proved that stromal cells targeting recombinant IL-2 in combination with ICB can be more effective in immunotherapy than ICB alone (80).

## The lipid metabolism of immune cells in TME

### Tumor-associated macrophages

**Figure 2:**
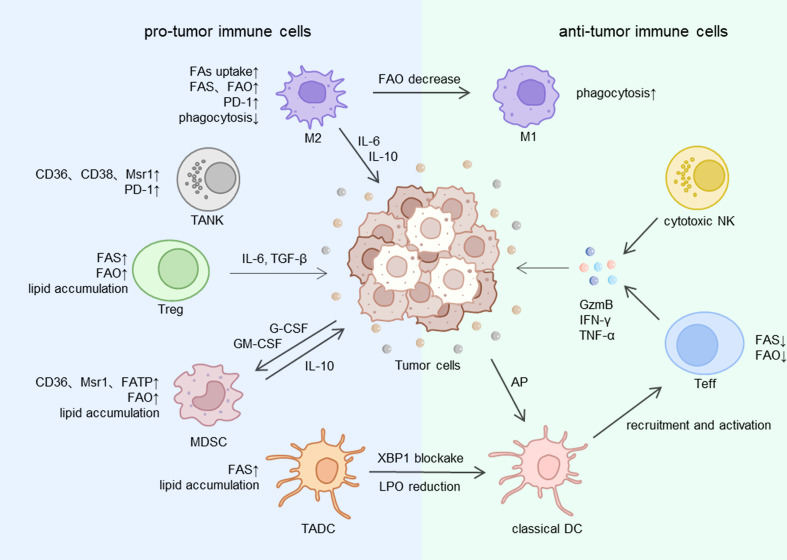
The lipid metabolism of immune cells in TME. FA, fatty acid; FAS, fatty acid synthesis; FAO, fatty acid oxidation; Msr1, macrophage scavenger receptor 1; FATP, fatty acid transport protein; XBP1, X-box binding protein 1; LPO, lipid peroxidation; AP, antigen presentation; IL, interleukin; TGF, tumor growth factor; G-CSF, granulocyte colony stimulating factor; GM-CSF, granulocyte macrophage colony stimulating factor; GzmB, granzyme B; IFN, interferon; TNF, tumor necrosis factor; M1, M1 macrophage; M2, M2 macrophage; NK, natural killer cell; TANK, tumor-associated natural killer cells; Teff, effective T cell; Treg, regulatory T cell; MDSC, myeloid-derived suppressor cell; DC,dendritic cell; TADC, tumor-associated dendritic cell.

TAMs have distinct characteristics from conventional macrophages. They infiltrate and settle down in the TME. Abundant studies have proved that TAMs have prominent pro-tumor activities, such as tumorigenesis, angiogenesis, spread and metastasis, drug-resistance and tumor immune evasion (81). TAMs are the most abundant immune cells in TME (82). The classically (M1) and alternatively (M2) activated macrophages phenotype are two different TAMs states of polarized activation (83). M2 macrophages are regarded as the main components of TAMs, which exhibit a tumor-promoting effect and are dedicated to forming an immunosuppressive microenvironment, while M1 macrophages show a tumor-inhibiting effect (84) (**Figure 2**). Consistently, the more amounts of M2 macrophages or total TAMs, the worse prognosis for patients with advanced gastric cancer (AGC) and hepatocellular carcinoma (85, 86). Lipid metabolism is closely involved in the selection of different polarization states in TAMs and ultimately leads to opposite effects in tumor (87).

Several processes of lipid metabolism like FA biosynthesis, uptake and storage are enhanced in TAMs (88). Free fatty acids are derived from extracellular uptake, *de novo* synthesis or release of LD from the cellular fatty acid pool. Research has indicated that the immunosuppressive phenotype of TAMs is induced by long-chain fatty acid metabolism, specifically unsaturated fatty acids (UFA) (89). Mechanically, to obtain enough energy and ensure the cellular survival, TAMs overexpress scavenger receptor CD36 and accelerate FAs transportation to accumulate lipid droplets and utilize FAO, enhanced FAO leads to a high rate of oxidative phosphorylation and STAT6 signaling pathway, which finally regulate gene transcription to determine the TAMs function (90). Given that CD36 is expressed extensively in other pro-tumor immune cells like regulatory T cells, the inhibition of CD36 may be a potential adjunct strategy to immunotherapy.

So, what factors will regulate FAO in TAMs? PPAR family has a strong link with FA metabolism in TAMs. PPARα, PPARδ and PPARγ are three major members of the PPAR family and they are involved in respective aspects of FA metabolism (91). S100A4 is a deciding factor for the subsets of TAMs: M2 macrophages display S100A4^+^ and M1 macrophages display S100A4^-^. S100A4 controls the upregulation of PPAR-γ during TAMs M2-like polarization, and then PPAR-γ upregulates CD36 to enhance FA uptake and FAO (92). In hepatocellular carcinoma, the deficiency of receptor-interacting protein kinase 3 (RIPK3) inhibits the caspase-1 mediated PPAR cleavage, and the reduction of PPAR clearance results in an increase in FAO and polarization of M2 TAMs (93). Intriguingly, the inhibition of caspase-1 could suppress tumor progression by promoting FAO and exhausting lipid droplets (94). The mechanism behind this contradiction still needs us to explore further. Moreover, another nuclear receptor liver X receptor (LXR), regarded as a cholesterol sensor and contributor to the loss of cholesterol intracellularly (95), play a pivotal role in TAMs gene expression. Activating LXR disrupts the mechanisms of TAMs to maintain immunosuppressive effects (96). M2 and M1 macrophages can be switched to each other in particular conditions. When changing the amounts of FAs or blocking FAO, M2 macrophages can be induced to transform into M1 type polarization (97) and the cancer progression can be prevented.

Compared to M2-like macrophages, M1-like macrophages express less PD-1 and escape from the effects of programmed cell death 1 ligand (PD-L1) (98). Therefore, we assume the combination of anti-PD-1 therapy towards malignant tumors with targeting lipid metabolism of TAMs might selectively strike M2-like macrophages without impairing M1-like macrophages to strengthen the effectiveness and sensibility of treatment.

### Tumor-infiltrating T lymphocytes

CD8^+^ and CD4^+^ cells are two types of TILs that are familiar to us and they play different roles in TME. The CD8^+^ clusters include effector T cells (Teff) that kill tumor cells directly, and CD4^+^ clusters include regulatory T cells (Treg), T follicular helper cells (Tfh) and T helper cells 1, 2, and 17 (Th1, Th2, Th17) (99), the function of Treg significantly contributed to the formation of immunosuppressive microenvironment (100, 101). Intratumor Tregs were found to be at high levels in some malignant tumors (102), one of the reasons is that both FAO and FAS are enhanced in Treg cells, thus Treg cells accumulate lipids and gain abundant energy to adapt to the undernourished environment in TME (103). By contrast, Teff tends to utilize glycolysis for more energy need (11), which will place Teff at a disadvantage in the hypoxia microenvironment (**Figure 2**).

Since activated T cells exhibit a desperate requirement for a high rate of FAO to maintain energy supply, the intracellular FAs content plays a crucial part in maintaining the levels of FAO in cells (104). FAs are known to be the essential materials for cells, and their deficiency in Teff inhibits cell proliferation and signal transduction so that the quantity of Teffs in tumor is low and cancer cells can’t be prevented efficiently. Another pro-tumor effect of FAs is that FAs catalyzed by ACC1 will fuel the differentiation of Tregs and Th17 (105). Tregs and Th17 have the synergistic effects in tumor-promoting with IL-6 and TGF-β secreted themselves (106). SREBP, a family of membrane-bound transcriptional regulators in ER, are mainly involved in FA and cholesterol synthesis. Three subsets are existing in human cells: SREBR1a, SREBP1c and SREBP2 (12). FAS is especially manipulated by SREBP1c (107). Studies showed that SREBP activity is simulated in intracellular Treg cells (108) and inhibited in Teff cells (109). To summarize, the levels of FA synthesis (modulated by SREBP1c) and FA oxidation (modulated by PPAR) are both elevated in Tregs to achieve a better immunosuppressive effect in tumor. And evidence showed that a high number of Tregs is associated with shorter overall survival and poor prognosis in several tumors like breast cancer or bladder cancer (110, 111).

The role of Th17 cells in tumors is like a coin with two sides, on one side they impair the immune response to the tumor, and on another side, they mediate anti-tumor immunity (112). More specifically, Th17 cells are divided into two categories according to whether they express CD5L (a regulator of lipid metabolism): nonpathogenic Th17 cells (CD5L^+^) and pathogenic Th17 cells (CD5L^-^). The former express immunosuppressive genes, while the latter display pro-inflammation genes (113). The deletion of CD5L remarkably reverses the function of Th17 cells by controlling lipid metabolism. For instance, CD5L deficiency leads to an elevated level of cholesterol ester and saturated fatty acid (SFA), and eventually induces nonpathogenic Th17 into pathogenic Th17 (114). Obviously, lipids act as important mediators in determining the phenotype and function of Th17 cells. In conclusion, changing lipid metabolism in T cell lines targeting genes, transcriptional regulators, or SFA content may become a potential strategy.

The regulation of lipid metabolism in T cells interacts with PD-1/PD-L1 expression. PD-1 is the major contributor to hinder tumor immune surveillance. High expression of PD-1 on cell surface is partly responsible for the depletion of CD8^+^ cells (115). Activation of PD-1 upregulates the expression of CPT1A and fatty triglyceride lipase (ATGL) so that T cells prefer to utilize FAO rather than glycolysis for energy supply (116, 117). We can learn that PD-1 expression reinforce FAO in Teffs. Interestingly, there is a study declaring that fenofibrate, a drug increases FAO of Teff cells, can reverse the poor effects of PD-1 blockade treatment (118). Therefore, we hypothesize that the enhancement of FAO at different levels might increase the PD-1 antibodies binding sites by increasing the expression of PD-1. The exact mechanism needs to be confirmed by abundant experiments in the future.

### Dendritic cells

DCs are the strongest antigen-presenting cells (119). DCs are composed of four main subtypes: classical DCs 1 (cDC1s), classical DCs 2 (cDC2s), plasmacytoid DCs (pDCs) and monocyte-derived DCs (moDCs). In addition to antigen presentation, another pivotal role of DC cells is to trigger the activation of cytotoxic T cells. cDC1s mediate anti-tumor immunity by activating cytotoxic CD8^+^ T cells, cDC2s activate anti-tumor CD4^+^ T cells but this process is restricted by Tregs or other immunosuppressive factors (120).

Even though the primary function of DC cells is inducing anti-tumor immune response in TME, a series of research has proved that the capability of anti-tumor of tumor-associated DC cells (TADCs) was destroyed *via* lipid accumulation (**Figure 2**). The augmentation of fatty acid synthesis relying on the ER and Golgi body serves as a key regulatory factor of TADC activation (121). High-rated FAS will eventually result in excessive lipids in the cytoplasm of TADCs. Abnormal lipid accumulation prevents the process of antigen-presenting and then weakens the tumor-inhibiting effects (120).

Exposure to the tumor environment results in several metabolic changes in TADCs. Firstly, TADCs overexpress the scavenging receptor macrophage scavenger receptor 1 (Msr1) that facilitates the perpetual uptake of fatty acids and cholesterol in TME (122). Excessive lipids are a sign of ER stress and oxidation damage because increased reactive oxygen species (ROS) mediate lipid peroxidation (123). Afterwards, the activation and cross-presentation to kill tumor cells are impaired. We have already mentioned there are many subtypes of DC cells, so grandly they possess distinct functions and metabolic pathways. It would explain why the phenomenon above was observed in cDC1 cells and cDC2 cells, but not in pDC cells (119). Furthermore, the mechanism of lipid peroxidation in TADCs to the ER stress response is mediated by the inositol-requiring protein 1α (IRE-1α) and its targeting X-box binding protein 1 (XBP1). XBP1 is regarded as the fuel of tumorigenesis and tumor cell progression. In ovarian cancer and triple-negative breast cancer, XBP1 is activated by the lipid peroxidation byproducts and aggregated lipids to promote the growth and dissemination of cancer cells (124). Therefore, the authors have proved that blockade or silencing of XBP1 makes TADCs regain the capability of immunostimulatory response and restriction towards tumor.

This brings us to the next question, what is the mechanism of excessive lipids driving these functional defects of TADCs? The pivotal step of TADCs to exhibit anti-tumor effects is the cross-presentation of tumoral antigens. Studies have established that electrophilic oxidatively truncated (ox-tr) lipids, one of the components of lipid bodies (LB), mediate the aggregation of heat shock protein 70 (HSP70) on the surface of large lipid droplets and then result in the deficiency of cross-presentation. It means that TADCs are unable to transport peptide MHC class I (pMHC) complexes to the cell surface as normal (125). In conclusion, modulating cross-presentation by reducing intracellular lipid aggregation of TADCs might become a potential therapeutic pathway. And there are experiments that exactly did so, deletion of *Atg5* (a key autophagy gene) in dendritic cells increased CD36 expression and lipid absorption, therefore, blocking CD36 in TADCs by regulating *Atg5* gene expression is a promising method to make a dent in lipids surplus (126, 127).

### Other immune cells

MDSCs have a powerful contribution to the formation of the immunosuppressive microenvironment of tumor (128) (**Figure 2**), which is controlled by several key factors like arginase-1 (ARG-1), COX2, TGFβ, iNOS, IL10 et al. But the detailed mechanisms are not completely same in two categories of MDSCs: polymorphonuclear (PMN-MDSCs) and monocytic (M-MDSCs) (129). In advanced gastric cancer, high content of MDSCs can be a conspicuous symbol of the awful prognosis (130). Similar to TADCs, lipid accumulation in cells also has a close relationship with MDSCs to achieve the immunosuppressive microenvironment. Exposure to G-CSF and GM-CSF secreted from tumor cells activates the STAT signal pathway and then elevates FA uptake and FAO in tumor infiltrating MDSCs (131). Continuous contact with exogenous UFAs like oleate but not free FAs synthesized within cells causes a regulatory phenotype induction in MDSCs and suppresses cytotoxic T cells’ proliferation and immune response (132). Exogenous lipids transporters like CD36, Msr1 and FATP are highly expressed on the surface of MDSCs to uptake lipids into cells and lead to a lipid-overloaded situation. Thus CD36 deficiency can diminish the useless lipid accumulation and immunosuppressive effect (9, 10). What’s more, previous studies have shown that FAO maintains the synthesis and secretion of tumor-promoting enzymes and cytokines, which is indispensable to the suppressed function of MDSCs. Thus, blockade of FAO wakens the Immune response tolerance mechanisms in MDSCs, prevents tumor progression, and even boosts adoptive T cells immunotherapy (131).

As a kind of cell easily underappreciated in tumor immunosuppressive environment, tumor-associated natural killer (TANK) cells have the important prognosis pertinence of the interaction with lipid metabolism and tumor cells (133)(**Figure 2**). In normal circumstances, NK cells express inhibitory receptors (KIRs, killer-cell immunoglobulin-like receptors) and activating receptors (NCRs, natural cytotoxicity receptors) on the surface, an appropriate proportion of them enables NK cells to monitor tumor cells in circulation through recognizing damage-associated molecular patterns (DAMPs) and pathogen-associated patterns (PAMPs) (134). However, studies in gastrointestinal cancer showed when NK cells infiltrate in the TME, their anti-tumor function is damaged on account of the unbalance between inhibitory and activating receptors (135). And the more advanced the tumor stage is, the more completely the function of TANK cells is destroyed.

One of the reasons accounts for the defects in TANK cells’ function and amount is that the immune checkpoint PD-1 high-express on both intratumoral TANK cells and circulating NK cells (136). It is possible that anti-PD-1 therapy can stop the decline of NK cells. Another reason is that oxidative stress caused by lipid peroxidation disrupts glucose metabolism and homeostasis. Nrf2 is the major antioxidant factor to protect TANKs from oxidative stress. Nrf2 agonist RTA-408 recovers TANK cells’ glycolysis and function, maintaining the effective lethality against tumor (137). Interestingly, with perioperative immunology becoming an emerging field recently, researchers suggested that tumor resection caused a postoperative lipid accumulation in NK cells in mice having beared colorectal cancer. This phenomenon is partially caused by gene expression of scavenger receptors like CD36, CD38 and Msr1 (138). These changes of NK cells attenuate the removal of residual tumor cells and increase the possibility of tumor recurrence. Targeting these changes may have an auxiliary effect after operations.

## Sensitivity and efficiency of combined immunotherapy and targeted lipid metabolism therapy

Immunotherapy is defined as a kind of cancer treatment strategy that enhances or restores immune function in the body through a variety of methods, including immune checkpoint blockade, adoptive T cell therapy, cancer vaccines and immune stimulants (139). In our review, we mainly talk about anti-PD-1 treatment which plays an important role in tumor immunotherapy. PD-1 is a well-known kind of inhibitory immune checkpoints. A series of research demonstrated PD-1 is found to be highly expressed in most immune cells including TAMs, T cells, DCs, NK cells and so on (98, 140–142). When PD-1 on Teffs is combined with PD-L1 expressed on tumor cells, the process will initiate the programmed cell death of T cells, thus tumor cells attack the anti-tumor immune response of activated T cells, achieving immune evasion. This is one of the main mechanisms for the formation of tumor immunosuppressive microenvironment. At present, PD-1/PD-L1 inhibitors have shown promising therapeutic effects in various cancers.

Low-response to immunotherapy is the biggest obstacle of immunotherapy and also the difficulty that must be broken through to realize the progress of immunotherapy. This low sensitivity is largely determined by the tumor cells themselves, such as low antigenicity, activation of the WNT pathway or MAPK pathway, inactivation of PTEN protein and the function of CDK proteins (143, 144). The regulation of lipid metabolism sometimes improves immunotherapy efficacy (**Table 1**) by indirectly influencing these processes. For example, one reason for low-sensitivity is the inactivation of the IFN-γ pathway in tumor cells. Teffs inhibit tumor cells by releasing IFN-γ, and tumor cells reduce Teffs by upregulating PD-L1 through IFN-γR-JAK-STAT pathway (157). Targeted lipid metabolism therapy promotes Teffs IFN-γ production, thereby promoting anti-PD-L1 therapy (153).

**Table 1:** The combination of lipid metabolism targeted therapy and immunotherapy.

Lipid metabolism targets	Metabolic agents	Immunotherapy combined	Immune cells	Mechanisms	References
FAO activator	Bezafibrate	Anti-PD-1	Teffs	Increase FAO to prevent cell death caused by FAO inhibition of anti-PD-1 therapy	(145–148)
ACAT1 inhibitor	avasimibe	CD19-CAR-T	Teffs	Block cholesterol esterification and facilitate TCR movement	(149, 150)
cPLA2-α inhibition	NA	Adoptive T cell transfer therapy	Teffs	Prevent the dysfunction and senescence of Teffs	(151)
CD36 inhibitor	NA	Anti-PD-1	Tregs	Block fatty acid intake and Inhibit the up-regulation of CPT by PD-1	(152)
FAO inhibitor	etomoxir	Adoptive T cell	MDSCs	Inhibit the infiltration of MDSCs and recruit the infiltration of Teffs	(131)
FATP2 blockade	Lipofermata	Anti-PD-L1	MDSCs	Up-regulate CD107a and reduce PD-L1 expression on tumor-infiltrating CD8^+^ T cells	(153)
PIM1 inhibitor	AZD1208	Anti-PD-L1	MDSCs	Inhibit FA uptake and FAO in MDSCs to decrease MDSCs and recruit Teffs	(154)
*Arf1* ablation	NA	Anti-PD-L1	DCs	Recruit and activate DCs and then increase Teffs’ infiltration	(155)
*HCK* ablation	NA	Anti-CTLA-4, anti-PD-1, anti-CD40	TAMs, DCs	Transform TAMs and DCs into inflammatory endotypes and recruit Teffs	(156)

NA, not available.

FAO, fatty acid oxidation; PD-1, programmed cell death protein 1; Teffs, effective T cells; ACAT, acyl-CoA cholesterol acyltransferase; CAR-T, chimeric antigen receptor T cells; TCR, T cell receptor; cPLA2-α, group IVA phospholipase A2; Tregs, regulatory T cells; CPT, carnitine palmitoyl transferase; MDSC, myeloid-derived suppressor cells; FATP, fatty acid transport protein; PIM, proviral insertion in murine malignancies; DCs, dendritic cells; CTLA-4, cytotoxic T lymphocyte antigen 4; TAMs, tumor-associated macrophages.

Moreover, lipid metabolites regulate immune checkpoints in various aspects. A large number of toxic lipid metabolism intermediates and by-products are produced and retained during high-efficiency lipid metabolism in various immune cells, which affect gene expression, transcription, modification and activation of immune checkpoints (158). *De novo* fatty acid synthesis promotes PD-1 expression in mature Tregs by protein geranylgeranylation and elevates PD-L1 expression in breast cancer by PD-L1 palmitoylation (108, 159). Considering that anti-PD-1 therapy still performs poorly in several patients and lipids play a pivotal role in immune cells’ function, the combination of lipid metabolism targeted therapy and PD-1 inhibitors is a novel idea to break through the bottlenecks in immunotherapy. Increasing research has proved that anti-PD-1 drugs can combine the following lipid metabolism targets at different levels to enhance anti-tumor immunity.

Treg cells are one of the most important members of intratumor immune cells with immunosuppressive effects. So blockade of lipid metabolism in Treg cells is the focus of research nowadays. Diverse patterns inhibiting Treg cells improve anti-tumor responses and prevent tumor metastasis (160, 161), but non-selectively suppression of T cells can lead to accidental damage of cytotoxic T cells and autoimmune caused by healthy Treg cell defects (152). Therefore, how to accurately kill Treg cells in tumors is a problem that needs to be solved at present. This prompts us to seek different biomarkers or signaling pathways between intratumoral Treg cells and normal T cells.

CD36 expression is up-regulated on the surface of both Tregs and cytotoxic T cells. Blocking CD36 effectively suppresses tumor growth (162) and prevents Tregs’ dysfunction. In a recent experiment, inhibiting CD36 in intratumor Tregs by genetic ablating or using CD36 monoclonal antibodies effectively retards tumor growth and has an auxiliary effect for anti-PD-1 treatment (152). CD36 deletion decreased several immune regulatory receptors’ expression but not PD-1, it could explain why the therapeutic effect of PD-1 was not impaired. Surprisingly, the body does not develop a severe autoimmune response in this way. Other studies about CD36 showed the over-expression of CD36 in intratumor effector T cells expose them to oxidative damage and ferroptosis, and the combination of anti-PD-1 therapy with inhibitors of CD36 or ferroptosis will produce greater immunotherapeutic benefits (163). The main reason why CD36 plays different roles in Tregs and effector T cells is that CD8+ T cells tend to rely on glycolysis for energy, while CD4+ T cells tend to obtain energy through FAO and OXPHOS in mitochondria (97). Therefore, Tregs promote mitochondrial adaptation to survive, but effector T cells suffer oxidative damage due to lipid accumulation (162).

We have mentioned above that FAS depends dreadfully on SREBP cleavage active protein (SCAP)/SREBP signaling pathway. Selectively block of this pathway to reduce FAS in Treg cells delays tumor growth and development in B16 melanoma mice. Mechanically, IFN-γ will be produced in quantity after deleting SCAP, and some processes like mevalonate metabolism and protein geranylgeranylation in over-expression of PD-1 rely on SCAP/SREBP pathway (108).

In addition to the dysfunction of Treg cells, the decreased and senescent cytotoxic T cell is another reason for tumor immune evasion. Senescent and dysfunctional T cells exhibit aberrant lipid metabolism and accumulation of lipids intracellularly. Scientists found elevated expression of group IVA phospholipase A_2_ (cPLA_2_-α) in both tumor cells and Treg cells, which is closely related to aging and lipid metabolism reprogramming in T cells *via* MAPK and STAT signaling pathways. Furthermore, cPLA_2_α inhibition prevents T cells from senescence and enhances immunotherapy such as adoptive T cell transfer therapy (151). Besides, using activator of FAO like bezafibrate could become an adjuvant for anti-PD-1 therapy. And bezafibrate achieves that mainly by facilitating the expression of CXCL9, CXCL10 from tumor cells and CXCR3 on intratumor Teffs (145–147).

There have also been studies on drugs that target lipid metabolism in other immune cells. The quantity of MDSCs is positively correlated with the degree of malignancy of tumor and the poor sensitivity of anti-PD-1 therapy. Combination of 5-fluorouracil and oxaliplatin decreases the amount of infiltrating MDSCs and then recruits CD8^+^ T cells in TME. However, there exists a disadvantage that PD-L1 expression on tumor cells is facilitated at the same time in this way (164). So it is necessary to explore how to reduce the side effects when diminishing MDSCs. The mechanism behind the connection between MDSCs and ICB resistance is that the gene expressions of CD8^+^ T cells in sensitive and insensitive patients are very different. CD8^+^ T cells in sensitive patients express effective and stimulating genes like *Fasl, Cd28, G2mb, Tnfsf4, lcos Prf1*, while in insensitive patients they express inhibitory and exhausted genes like *Pdcd1.* Blockade of PIM1 (a serine/threonine kinase) with drugs or genetic deletion inhibits FA uptake and FAO in MDSCs through PPARγ-mediated pathway, which finally decreases the quantity of MDSCs, recruits CD8^+^ T cells and ameliorate ICB resistance (154).

The removal of *Arf1* (a mediator of lipid metabolism to maintain the abundance of tumor stem cells) induces DC cell activation and activated DC cells increase CD8^+^ T cells’ infiltration to kill tumor cells. Mechanically, *Arf1* ablation in tumor stem cells induces mitochondrial disorder and the release of DAMPs, which recruit and activate DCs at tumor sites. Therefore *Arf1* ablation and PD-1 blockade have a synergistic effect (155). Besides, another study has demonstrated that genetic ablation of myeloid-specific hematopoietic cell kinase (*HCK*) boosts the therapeutic effect of anti-CTLA-4, anti-PD-1 and anti-CD40 immunotherapy. The deficiency of *HCK* transforms TAMs and DCs into inflammatory endotypes and then recruits CD8^+^ T cells (156).

Nowadays, ferroptosis in tumor cells is an emerging field for tumor treatment strategy. Ferroptosis, an iron-dependant programmed cell death, is induced by lipid peroxides retention and ROS (165). PKCβII-ACSL4 pathway is the main contributor to inducing tumor ferroptosis through sensing and exaggerating lipid peroxides. Ferroptosis is partially associated with immunotherapy and patients with high ACSL4 display a high sensitivity to anti-PD-1 therapy. So enhanced PKCβII-ACSL4 pathway can improve the efficacy of immunotherapy by promoting ferroptosis (166). Consistently, there are a series of experiments showing that manipulating ferroptosis in tumor cells, such as low dose arachidonic acid (AA), inhibition of CAMKK2 or AMPK-NRF2 pathway, inhibition of ALG3 et al. (167–169), can boost the efficacy of anti-PD1 therapy.

In general, FAS and FAO are involved in many aspects of tumor cells and immune cells, including energy acquisition, raw materials of membrane structure, regulation of signaling pathways, formation of the immunosuppressive microenvironment and mediating ferroptosis. Manipulation of lipid metabolism at different levels, such as regulating enzymes, transcription factors or gene expression involved in lipid metabolism, can play a synergistic role with ICB-induced anti-tumor immunotherapy. The common mechanism of most combination therapy is the recruitment and activation of CD8^+^ T cells. Moreover, some lipid metabolism-related genes can predict the prognosis of anti-PD-1 therapy (170), which may alleviate the problem of low response rates. In order to figure out the mechanisms or pathways involved and improve the effectiveness and sensitivity of immunotherapy, abundant experiments are needed to be warranted.

## Conclusion and perspectives

The combination of lipid metabolism targets in either immune cells or cancer cells therapy and ICB-induced immunotherapy exhibits the potential to be a novel cancer treatment strategy. This idea was put forward based on two facts. One is that the inhibition of PD-1/PD-L1 therapy encounters the low-response rate in certain patients, which is closely linked with the exhaustion of CD8^+^ T cells, that’s why the major mechanism for many combination therapies is recruitment and activation of Teffs. And the other one is that lipid metabolism reprogramming not only plays a vital role in immune cells and the formation of the immunosuppressive microenvironment, but is also involved in the expression of PD-1. Lipid metabolism reprogramming plays contradictory roles in the immune microenvironment of tumors. In situations of intense competition for nutrients, immune cells use lipids as fuel to mount immune responses. Lipids and cholesterol regulate different immune cells cytotoxic effects, for example, cholesterol enhances cytotoxic effects by restoring the IFN-γ production of iNKT cells. However, Excessive lipid accumulation and LD formation damage T cells or DC cells and induce macrophages and MDSC cells to differentiate into pro-tumor subtypes (171). Although various preclinical trials of this combination therapy achieve convincing and satisfactory results, the systematic and comprehensive mechanisms of lipid metabolism in coordinating anti-tumor or pro-tumor effects of distinct immune cells are still not fully validated. Further, the pathway through which alteration of lipid metabolism reverses the poor sensitivity of anti-PD-1 also requires to be explored through more related experiments. Take FAO as an example, increased FAO is a double-edged sword. On one side, enhanced FAO promotes the expression of PD-1 and polarization of M2, which accelerates tumor progression. On the other side, treatment increasing FAO boosts the effect of anti-PD-1 therapy. So how to balance the level of lipid metabolism manipulation, the specific mechanism and strategy deserve further studies, this may become the novel breakthrough encountering the obstacles of immunotherapy.

## Author contributions

ZC and DW conceived the concept, DW and QY wrote the manuscript, ZC and HG revised this review. All authors contributed to the article and approved the submitted version.

## Funding

This work was supported by the National Natural Science Foundation of China (81972598), Health Commission of Zhejiang Province (WKJ-ZJ-1803), Natural Science Foundation of Zhejiang Province (LQ20H160064, LY19H160004) and Fundamental Research Funds for the Central Universities (2020FZZX003-02-12, 2021FZ203-02-08).

## Conflict of interest

The authors declare that the research was conducted in the absence of any commercial or financial relationships that could be construed as a potential conflict of interest.

## Publisher’s note

All claims expressed in this article are solely those of the authors and do not necessarily represent those of their affiliated organizations, or those of the publisher, the editors and the reviewers. Any product that may be evaluated in this article, or claim that may be made by its manufacturer, is not guaranteed or endorsed by the publisher.
